# Distinct neurogenic progenitor cell populations balance cell type production in the embryonic mouse retina

**DOI:** 10.64898/2026.08.03.738451

**Published:** 2026-08-04

**Authors:** Henry L. Bushnell, Constance L. Cepko

## Abstract

Mammalian vision depends on the reliable production of over 100 neural cell types from multipotent retinal progenitor cells. How so many cell types are generated in the correct proportions throughout the retina remains incompletely understood. Focusing on the early embryonic period in mice, we found that retinal neurogenesis is organized through discrete neurogenic progenitor cell (NPC) populations with distinct fate biases. NPCs expressing *Galanin* primarily produce retinal ganglion cells and amacrine cells, whereas *Olig2* -expressing NPCs produce cones and horizontal cells in the same temporal window. Clonal analysis revealed that these two fate-biased NPC populations arise predominantly from asymmetric, renewing progenitor cell divisions that produce one fate-biased NPC daughter cell. Differential Notch signaling regulates the production of these NPC populations, with *Galanin* ^+^ and *Olig2* ^+^ NPCs representing Notch ^High^ and Notch ^Low^ states, respectively. Manipulating Notch signaling was sufficient to toggle their production. The expression of Notch signaling components across NPCs is consistent with a lateral inhibition mechanism, in which fate-biased NPCs promote neighboring cells to adopt a complementary fate-biased NPC state. These findings suggest that the robust production of diverse retinal cell types is achieved through local feedback that balances discrete, fate-biased NPC populations.

## Full Text Availability

The license terms selected by the author(s) for this preprint version do not permit archiving in PMC. The full text is available from the preprint server.

